# Ammonium-tagged ruthenium-based catalysts for olefin metathesis in aqueous media under ultrasound and microwave irradiation

**DOI:** 10.3762/bjoc.15.16

**Published:** 2019-01-17

**Authors:** Łukasz Gułajski, Andrzej Tracz, Katarzyna Urbaniak, Stefan J Czarnocki, Michał Bieniek, Tomasz K Olszewski

**Affiliations:** 1 Apeiron Synthesis SA, Duńska 9, 54-427 Wrocław, Poland; 2 Wrocław University of Science and Technology, Faculty of Chemistry, Wybrzeże Wyspiańskiego 29, 50-370 Wrocław, Poland

**Keywords:** catalysis, green chemistry, microwave, *N*-heterocyclic carbene, olefin metathesis, ruthenium, ultrasound

## Abstract

The influence of microwave and ultrasonic irradiation on the performance of ammonium-tagged Ru-based catalysts in olefin metathesis transformations in aqueous media was studied. Differences in the catalytic activity in correlation with the nature of the present counter ion and the size of the *N*-heterocyclic carbene (NHC) ligand were revealed. The presented methodology allows for preparation of a variety of polar and non-polar metathesis products under environmentally friendly conditions.

## Introduction

Olefin metathesis is well established as a powerful transformation used for effective and elegant creation of new carbon–carbon double bonds [1–2]. The development of commercially available, stable and effective catalysts for that reaction [3–6] made possible for its wide application not only in academia but also in industry [7–12]. However, there is still a large interest in improving the catalytic activity of the existing Ru-based metathesis catalysts as there is no universal catalyst for all the metathesis transformations. This is especially true for olefin metathesis reactions carried out with the use of green solvents, for which there is currently an increasing demand, especially in industrial practice, as a replacement for those with major regulatory issues such as chlorinated (dichloromethane, 1,2-dichloroethane) or aromatic solvents (toluene, benzene) [13–16]. In that aspect olefin metathesis in aqueous media appears to be an interesting alternative, especially in the case of preparation of biologically important molecules [17–20] as well as of highly polar compounds. Thus far, several strategies were applied to facilitate olefin metathesis in water including the development of specially designed water-soluble catalysts [21–28], addition of organic solvents [29–31], or use of additives such as for example calixarenes or cyclodextrins [32–33], chloride salts [34], vitamin E-based amphiphiles [35], dodecyltrimethylammonium bromide (DTAB) [36], polymerised cyclooctadiene (COD) and cyclooctene (COE) [37], sodium dodecyl sulphate (SDS) [38] or DL-α-tocopherol methoxypolyethylene glycol succinate solution (TPGS-750-M) [39], to improve the solubility of reacting species and/or performance of the catalyst. Recent progress in the flourishing field of micellar catalysis and the use of surfactants that self-aggregate in water into micelles in which the hydrophobic core provides an environment for effecting homogeneous reactions between organic molecules has been reviewed by Scarso et al. [40] and very recently by Lipshutz and co-workers [41]. Worth mentioning are also reports of heterogenous and recyclable catalysis able to mediate metathesis in aqueous media [42–45]. Although the aforementioned examples show a significant progress in the olefin metathesis in aqueous media, some limitations such as complex structure of the tailored catalysts and thus difficulties associated with their synthesis, or the need to use additives or co-solvents to improve the solubility of reacting species, still remain. Therefore, further development of catalytic systems would provide a complimentary extension to the scope of this interesting transformation.

Furthermore, in the continuous search for new sustainable protocols for chemical reactions to induce new reactivates or reduce the energetic cost of the processes, the replacement of mechanical mixing and/or heating of the reacting species with microwave (μW) [46–48] and ultrasonic irradiation (US) [49–55] appears as a promising approach. Both methods were shown in the past to be responsible for shortening the reaction time, increasing the reaction yield or even favour the formation of the desired product when compared to traditional protocols [56–57]. In the case of olefin metathesis, examples of application of those techniques are well documented for organic solvents [58–66], surprisingly, examples describing reactions in aqueous media are scarce and thus merit further investigation [67–68].

In line with our ongoing research on synthesis of catalysts for olefin metathesis and to expand the utility of ammonium-tagged ruthenium-based catalysts [69–76], herein we present the use of such catalysts for olefin metathesis in aqueous media promoted by microwave and ultrasound irradiation.

## Results and Discussion

**Figure 1:**
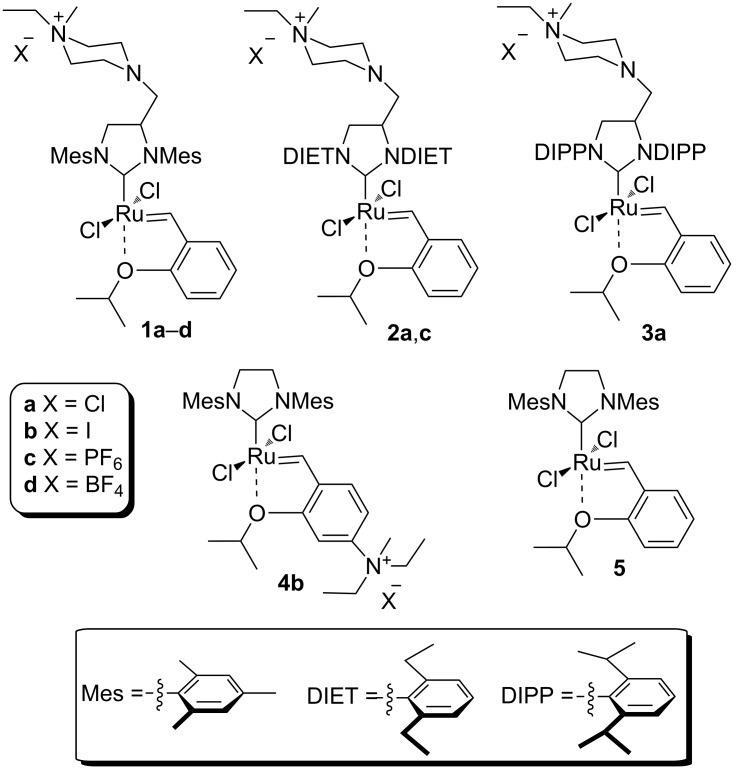
Structures of the Ru-based catalysts used in this study.

The structures of the catalysts **1**–**5** used in this work are depicted in Figure 1.

Catalyst **1b** was prepared by alkylation of the non-ionic tertiary amine-containing analogue with methyl iodide [71]. Complexes **1c**,**d** were prepared from their commercially available corresponding chloride salt **1a** [72] by exchange of the Cl^−^ counter-ion to PF_6_^−^ or BF_4_^−^ [76]. The exchange was performed in water, and after addition of NH_4_PF_6_ or NH_4_BF_4_ the formed catalysts were collected by filtration. Catalyst **2c** was prepared from the new complex **2a** using a similar procedure (see Supporting Information File 1 for details). The complexes **3a** [75] and **5** are commercially available and catalyst **4b** was obtained according to a literature procedure from commercially available Grubbs 2nd generation catalyst through ligand exchange [68]. In general, the solubility of the catalysts containing Cl^−^ as counter ion in water is good (e.g., 50 mg mL^−1^ for **1a** or **3a**) whereas for those with I^−^ as counter-ion is much lower (e.g., 4.0 mg mL^−1^ for **1b**). In turn catalysts bearing PF_6_^−^ or BF_4_^−^ as counter ions are not soluble in water [71].

We have started our study with the comparison of the catalytic activity of complex **4b**, having the ionic tag attached to the benzylidene ligand, with that of catalyst **1a**, bearing an ionic tag placed on the *N*-heterocyclic carbene (NHC) fragment. As model reactions we have selected the ring-closing metathesis (RCM) of the water-soluble substrate **6**, the homometathesis of alcohol **8**, and more challenging, the cross metathesis (CM) between alcohol **8** and the electron-deficient cross partner methyl acrylate (**10**, Table 1).

**Table 1:** Effect of microwave (μW) and ultrasound (US) irradiation on RCM, homometathesis and CM in water mediated by complexes **1a** and **4b**.

Entry	Substrate	Product	Ru complex	Classical conditions^a^	US^a^	µW^a^

1^b^	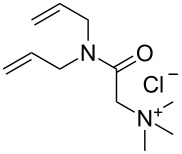 **6**	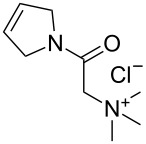 **7**	**1a**	52	63	48
2^b^			**4b**	48	59	55
3^c^	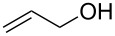 **8**	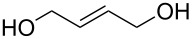 **9**	**1a**	81 (78)	73 (60)	64 (68)
4^c^			**4b**	77 (88)	38 (66)	75 (84)
5^c^	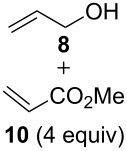	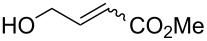 **11**	**1a**	69 (74)	71 (79)	81 (88)
6^c^			**4b**	35 (45)	71 (80)	80 (86)

^a^Conversion and selectivity (in parentheses, referring to the formation of an aldehyde, having a signal at 9.60 ppm, resulting from double bond migration) have been determined based on ^1^H NMR. ^b^Reaction conditions: D_2_O, catalyst (1 mol % Ru), *c* 0.1 M, 36 °C, 2 h. ^c^Reaction conditions: D_2_O, catalyst (5 mol % Ru), *c* 0.1 M, 36 °C, 2 h.

All reactions were run at 36 °C in D_2_O promoted either by microwave (µW) or ultrasound (US) irradiation, and for comparison purposes also with standard magnetic stirring. In the case of the RCM (Table 1, entries 1 and 2) both tested catalysts (1 mol %) under classical conditions exhibited similar activities with **4b** being slightly less active (52 vs 48%, respectively). The reaction performed under ultrasound irradiation proved to be ca. 10% more productive with both catalysts compared to the classical conditions. On the other hand, microwave irradiation turned out to be less effective leading to a drop in the reaction yield for **1a** (48%) and a slightly increased yield in the case of **4b** (55%). In the homometathesis reaction of allyl alcohol **8** (Table 1, entries 3 and 4) both catalysts (5 mol %) produced the desired product again with quite similar yields under classical conditions. However, the use of microwave or ultrasound irradiation promoted the undesired isomerisation of the C=C bond, thus lowering the yields of the desired product **9** (Table 1, entries 3 and 4). This result is in agreement with the known fact that in protic solvents ruthenium hydrides are formed leading to isomerisation byproducts [66]. Finally, we were pleased to see that the use of ultrasound or microwave irradiation were beneficial for the CM of alcohol **8** with methyl acrylate (**10**, Table 1, entries 5 and 6) resulting not only in increased conversion but also reducing the amount of the unwanted product of self-metathesis of **8**.

In general, the results obtained with catalysts **4b** and **1a** were comparable. However, we expected that **1a** should be much more effective because it remains tagged after the initiation step. This unexpected catalytic activity might be due to the fact that catalysts **4b** and **1a** have different counter ions and therefore we decided to examine if there is an influence of counter ions on the catalytic activity. To achieve this we used analogues of **1a** bearing different counter ions (**1b**–**d**) and also included catalysts having differently sized NHC ligands (**2a**,**c**). For testing the catalysts performances, we selected the RCM of the water-soluble substrate **12** (Table 2).

**Table 2:** Effect of the counter ion and substituents size of the NHC ligand in catalysts **1b**–**d**, and **2a**,**c** on their efficiency in the RCM of substrate **12** in water under μW and US irradiation.^a^

Entry	Substrate	Product	Ru	Classical conditions^b^	US^b^	µW^b^

1	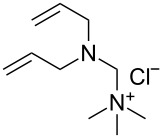 **12**	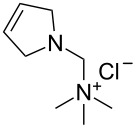 **13**	**1a**	33	41	58
2			**1b**	13	53	77
3			**1c**	53	49	81
4			**1d**	35	46	72
5			**2a**	54	12	51
6			**2c**	33	32	61
7			**5**	3	1	1

^a^Reaction conditions: D_2_O, Ru catalyst (0.25 mol %), *c* 0.2 M, 36 °C, 2 h. ^b^Conversions determined based on NMR.

Under the reaction conditions the classical catalyst **5** (0.25 mol %) was not soluble resulting in poor yields and justifying the use of modified catalysts. For the ammonium NHC-tagged catalysts (0.25 mol %), the use of microwave irradiation was more productive than ultrasound treatment. This effect was most pronounced in the case of catalysts with low solubility in water such as **1b** or the insoluble catalysts **1d** and **2c**. Additionally, under classical conditions, we observed a higher activity of catalyst **2a** with Cl^−^ as counter ion and a larger NHC ligand when compared to **1a**. In turn, the use of US and μW had an inverse effect on that reaction furnishing lower yields of the product in the case of **2a** when compared with **1a**. In case of catalysts bearing a large hexafluorophosphate counter ion (PF_6_^−^; **1c** and **2c**) an increase of the NHC’s size had a negative effect on the catalyst performance (Table 2, entries 3 and 6, respectively).

Examining further the influence of the steric hindrance of the NHC ligand we tested complexes **1a**, **2a** and **3a** (1 mol %) all with Cl^−^ as counter ion in the RCM of polar substrate **6** (Table 3).

**Table 3:** Effect of the size of the NHC ligand in the catalysts **1a**, **2a**, and **3a** on their catalytic efficiency in the RCM of substrate **6** under μW and US irradiation.^a^

Entry	Substrate	Product	Ru	Classical conditions^b^	US^b^	µW^b^

1	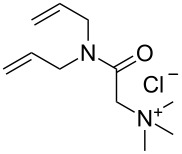 **6**	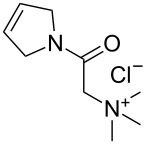 **7**	**1a**	61	48	51
2			**2a**	43	38	36
3			**3a**	22	61	39

^a^Reaction conditions: D_2_O/iPrOH 2:1 (v/v), Ru catalyst (1 mol %), 36 °C, 2 h. ^b^Conversions determined based on NMR.

In order to maintain homogeneity of the reaction mixture addition of isopropanol (iPrOH) was necessary. Under the applied conditions the activity of the tested complexes decreased with increasing size of the NHC ligand. This result suggests that a fast propagation ensured by a smaller carbene ligand rather than robustness ascribed to larger catalysts is a prerequisite for the efficient metathesis in homogeneous aqueous conditions. Except in the case of catalyst **3a**, exhibiting the lowest activity under classical conditions, we noted a positive effect of US increasing the reaction yield from 22 to 61%.

Finally, we have tested the influence of μW and US irradiation on the RCM of lipophilic substrates **14**, **16** and **18** in water (Table 4).

**Table 4:** Effect of microwave (μW) and ultrasound irradiation (US) irradiation on the RCM of lipophilic substrates in water.^a^

Entry	Substrate^b^	Product^b^	Ru	Classical conditions^c^	US^c^	µW^c^

1	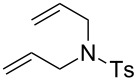 **14**	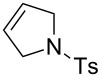 **15**	**1a**	21	6	17
2			**1b**	95	95	97
3			**1c**	83	92	93
4			**1d**	93	80	89
5			**2a**	20	6	12
6			**2c**	73	91	97
7			**5**	49	63	96

8	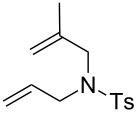 **16**	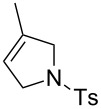 **17**	**1a**	24	3	11
9			**1b**	82	29	46
10			**1c**	96	73	74
11			**1d**	90	25	55
12			**2a**	12	2	7
13			**2c**	96	95	83
14			**5**	88	78	77

15^d^	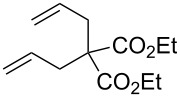 **18**	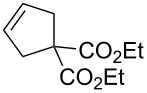 **19**	**1a**	3	2	3
16^d^			**1b**	78	15	56
17^d^			**1c**	93	27	64
18^d^			**1d**	78	11	57
19^d^			**2a**	3	2	2
20^d^			**2c**	93	37	67
21^d^			**5**	80	69	50

^a^Reaction conditions: H_2_O, Ru catalyst (0.5 mol %), *c* 0.2 M, 36 °C, 2 h. ^b^Ts: *p*-toluenesulfonyl. ^c^Conversions determined based on GC. ^d^Reaction conditions: H_2_O, Ru catalyst (1.0 mol %), *c* 0.2 M, 36 °C, 2 h.

Regardless of the conditions and substrate used, the lowest yields were observed for the water-soluble complexes **1a** and **2a**. This is most probably due to the reduced stability of those catalysts in aqueous medium and additionally to their limited contact with the substrates, being in a different phase (organic layer), and in a process that resembles more to a “heterogeneous reaction”. In turn, reactions with the use of catalysts **1b**, **1c**, **1d** and **2c** with much lower solubility in water gave significantly better results. With only two exceptions the use of μW or US irradiation provided poorer results when compared to the classical conditions. Only catalysts **1c** and **2c** bearing PF_6_^−^ as counter ion performed better with both, μW and US irradiation. However, this effect was observed only for the simplest substrate **14**. The results collected in Table 4 can be explained by the fact that the reactions actually occurred under heterogeneous conditions between water-insoluble components at the water–reagents phase boundary [26,77–78]. Such conditions can imply a positive impact on the rate of a reaction and are the result of a “hydrophobic effect” [78–79]. This phenomenon, mentioned by Sharpless and co-workers in their seminal paper [78] is not well understood yet [80]. Jung and Marcus postulated a trans-phase hydrogen bonding from water OH groups to H-bond acceptor sites of organic reactants contributing to a stabilisation of organic transition states enables the on-water catalysis [77]. Ben-Amotz et al. demonstrated that the effect of the water OH groups depends either on the surface area involved or on the electrostatic nature of the surface itself [81]. Additionally, the packing density of supramolecular clusters of water created by strong intermolecular hydrogen bonds may also play a key role. Indeed, various effects may be depending on the solubility of the reactants in water [82–83]. The hydrophobic and water molecules stay in minimal contact between each other because a sphere of water molecules is formed around the non-polar components resulting in higher (local) concentration and higher pressure in water [26,79]. The application of US and μW irradiation could, to some extent, disturb the “hydrophobic effect” and thus may explain the less satisfactory results of the reactions using those techniques compared to those obtained under classical conditions.

## Conclusion

We have examined the effect of microwave and ultrasonic irradiation on a range of different olefin metathesis transformations in water catalysed by ammonium-tagged Ru-based catalysts. It was noted that placing the water solubilising ionic tag on the NHC ligand gives catalysts with improved catalytic activity and more suitable for reactions in water than those having an ionic tag on the benzylidene part. In general, a more prominent positive effect of microwave irradiation on the reaction outcome compared to ultrasound was observed. This effect was shown in a CM reaction, where an improvement in the reaction yield and selectivity was noted, as well as in the RCM of water-soluble substrates. In reactions with lipophilic substrates the solubility of the tested catalysts had a crucial influence on the reaction outcome. In turn, the use of microwave and ultrasonic irradiation did not have a positive effect on the reaction productivity. In contrast, catalysts that are sparingly or even insoluble in water gave better results that were explained by the “hydrophobic effect”.

## Supporting Information

File 1 Experimental procedures and characterisation data for all previously unreported compounds.
