# Supplementary material for: Ammonium-tagged ruthenium-based catalysts for olefin metathesis in aqueous media under ultrasound and microwave irradiation
Source: Beilstein J Org Chem. 2019 Jan 17;15:160–6. doi: 10.3762/bjoc.15.16 (PMC6350890; doi:10.3762/bjoc.15.16)

BEILSTEIN JOURNAL OF ORGANIC CHEMISTRY

Supporting Information

for

Ammonium-tagged ruthenium-based catalysts for olefin metathesis in aqueous media under ultrasound and microwave irradiation

Łukasz Gułajski, Andrzej Tracz, Katarzyna Urbaniak, Stefan J. Czarnocki, Michał Bieniek and Tomasz K. Olszewski

Beilstein J. Org. Chem. **2019,** *15,* 160–166. doi:10.3762/bjoc.15.16

Experimental procedures and characterisation data for all previously unreported compounds

License and Terms: This is a supporting information file under the terms of the Creative Commons Attribution License (http://creativecommons.org/licenses/by/4.0). Please note that the reuse, redistribution and reproduction in particular requires that the authors and source are credited.
The license is subject to the *Beilstein Journal of Organic Chemistry* terms and conditions: (https://www.beilstein-journals.org/bjoc)

Table of contents

1. General information S1

2. Synthesis and of catalysts 2a and 2c S2

2.1 Synthesis of catalyst **2a** S2

2.2 Synthesis of catalyst **2c** S9

3. Olefin metathesis reactions S11

3.1 General procedure for metathesis under classical conditions S11

3.2 General procedure for metathesis under ultrasound irradiation S11

3.3 General procedure for metathesis under microwave irradiation S11

4. Synthesis and characterization of compounds 6 and 7: S12

4.1 Synthesis of compound **6** S12

4.2 Characterisation of compound **7** S13

1. General Information

All reagents for substrate and catalysts synthesis were purchased from Sigma and used without further purification. Deuterium oxide (99.9% atom D) was purchased from Sigma.

NMR spectra were recorded on Burker Avance^{TM} 600 MHz spectrometer in CD_2Cl_2, $CDCl_3$ or D_2O. GC analyses were performed on Perkin Elmer Clarus 680 with FID detector and GL Sciences InertCap 5MS/NP capillary column. High resolution mass spectra were recorded on MaldiSYNAPT G2-S.

Reactions under ultrasounds were conducted in Ultron U-20 water bath, equipped with heater and electronic temperature control.

Reactions under microwave irradiation were conducted in CEM Mars 6^{TM} equipped with MarsXpress Teflon tubes and IR bottom temperature sensor.

Catalysts **1a**–**d** and **3a** were previously reported by us[1-4]. Catalysts **1a**, **1d**, **3a** are commercially available from STREM Chemicals Ltd. Catalyst **5** is available from Sigma-Aldrich.

All metathesis substrates and products except **6** and **7** are known compounds. The identity of metathesis products previously reported by us and affirmed by NMR spectroscopy was confirmed by NMR and comparison of GC retention times.

---

[1] J. Pastva, K. Skowerski, S. J. Czarnocki, N. Zilkova, J. Cejka, Z. Bastl, H. Balcar, *ACS Catal.*, 2014, **4(9)**, 3227.
[2] K. Skowerski, C. Wierzbicka, G. Szczepaniak, Ł. Gułajski, M. Bieniek, K. Grela, *Green Chem.*, 2012, **14**, 3264.
[3] K. Skowerski, J. Pastva, S. J. Czarnocki, J. Janoscova, *Org. Process Res. Dev.*, 2015, **19(7)**, 872
[4] A.Tracz, A. Gawin, M. Bieniek, T. K. Olszewski, K. Skowerski, *New J. Chem.*, 2018, **42**, 8609

2. Synthesis of catalysts 2a and 2c

2.1 Synthesis of catalyst 2a

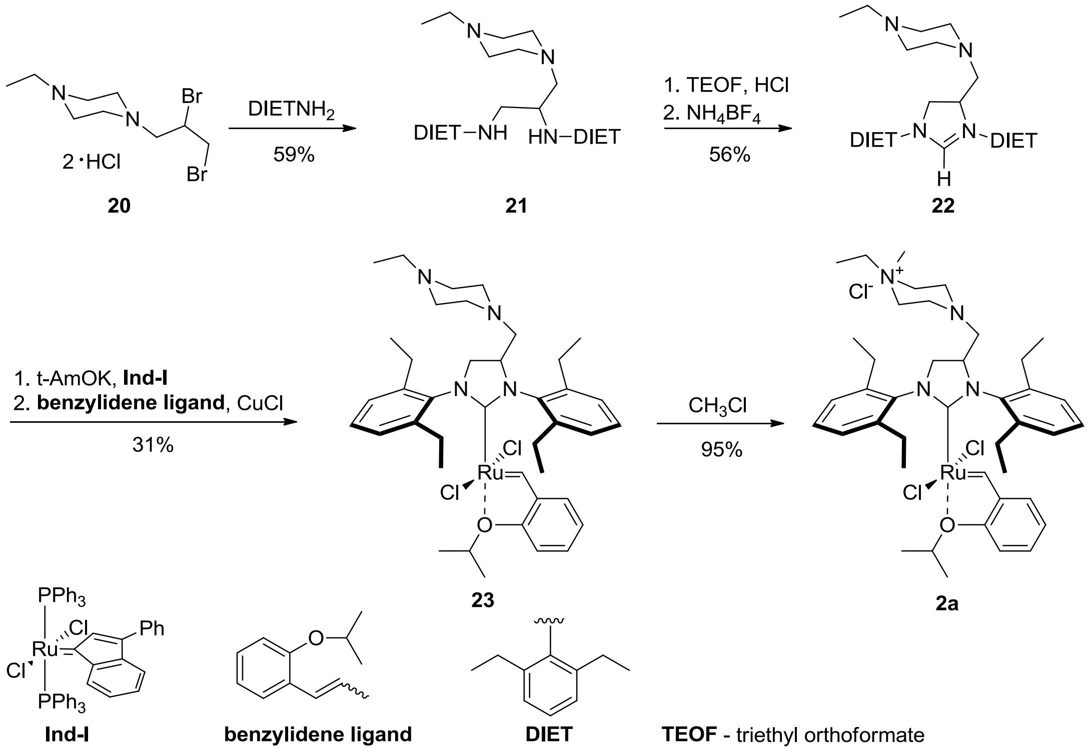

Scheme S1: Synthesis of catalyst **2a**.

2.1.1 *N*[1],*N*[2]-Bis(2,6-diethylphenyl)-3-(4-ethylpiperazin-1-yl)propane-1,2-diamine (21)

1-(2,3-Dibromopropyl)-4-ethylpiperazine hydrochloride (**20**, 124 g, 320 mmol) was added to 2,6-diethylaniline (335 g, 2243 mmol). The mixture was stirred for 20 h at 125 °C. Next, the reaction mixture was cooled to room temperature and a 4 M aqueous solution of sodium hydroxide was added (400 ml). The mixture was extracted with dichloromethane. The organic layer was washed with water and the solvent was evaporated. The remaining 2,6-diethylaniline was removed under reduced pressure and the crude product was purified using column chromatography (5% AcOEt in c-hex to 40% AcOEt in c-hex as eluent). The product was obtained as dark-yellow oil (86g, 59% isolated yield).

^{1}H NMR (600 MHz, CDCl$_3$) δ ppm: 7.13-7.05 (m, 4H), 7.03-6.95 (m, 2H), 3.76-3.68 (m, 1H), 3.54-3.34 (m, 1H), 3.23-3.10 (m, 1H), 3.09-3.03 (m, 1H), 2.93-2.24 (m, 20H), 1.33 (t, *J* = 7.8, 7H), 1.24 (t, *J* = 7.2, 6H), 1.18-1.11 (m, 3H).

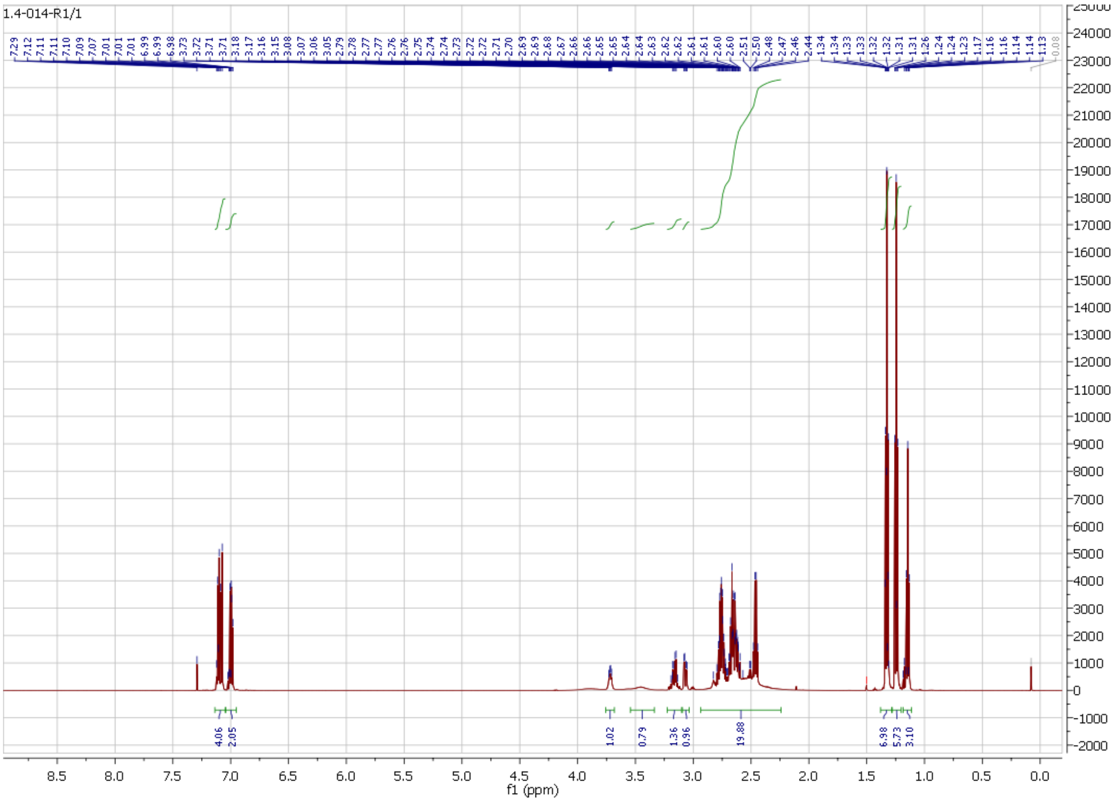

^{13}C NMR (150 MHz, CDCl$_3$) δ ppm: 145.32, 145.26, 145.25, 136.76, 136.06, 135.14, 126.75, 126.65, 126.63, 122.69, 122.56, 122.00, 65.32, 62.65, 55.30, 54.10, 53.64, 52.92, 52.53, 52.42, 48.57, 48.15, 24.91, 24.59, 24.33, 15.16, 15.01, 14.52, 12.06.

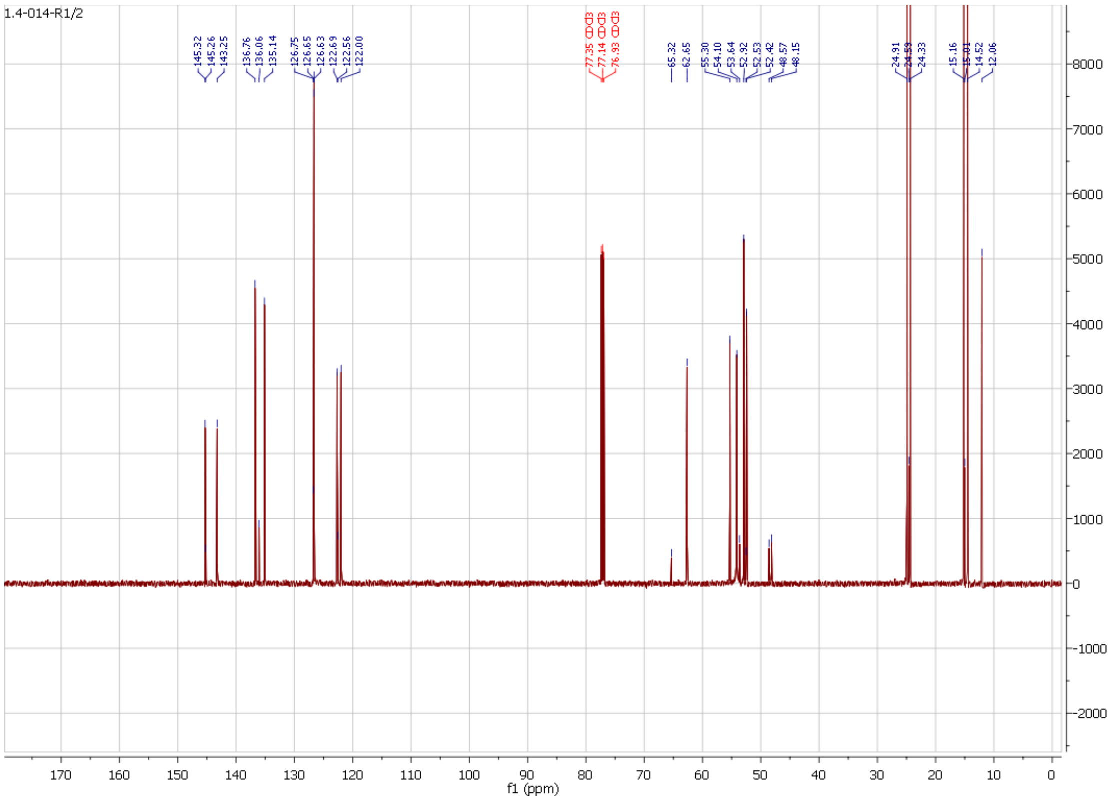

2.1.2 1,3-Bis((2,6-diethylphenyl)-4-((4-ethylpiperazin-1-yl)methyl)-4,5-dihydro-1*H*-imidazol-3-ium tetrafluoroborate (22)

Compound **21** (50 g, 111 mmol) was dissolved in orthoformate (164 g, 1109 mmol) and 4 M hydrochloride solution in 1,4-dioxane (55.5 ml, 222 mmol). The mixture was stirred for 2.5 h at 105 °C. Then, the solvents were removed and water (500 ml) was added followed by the addition of NH_3 (28% in water, 22.24 ml, 333 mmol). Insoluble material was filtered off and rejected. A saturated solution of NH_4BF_4 in water (23.26 g, 222 mmol) was added to the filtrate and the product extracted with dichloromethane (3 x 80 ml). Next, *tert*-butyl methyl ether was added and dichloromethane slowly evaporated. The precipitated product (white solid) was filtered off, washed with *tert*-butyl methyl ether and dried under reduced pressure (34.05 g, 56% yield).

^{1}H NMR (600 MHz, $CDCl_3$) δ ppm: 8.09 (s, 1H), 7.40-7.35 (m, 2H), 7.25-7.11 (m, 4H), 5.20-5.10 (m, 1H), 4.73 (t, *J* = 12.0 Hz, 1H), 4.05 (dd, *J* = 12.6, 9.0 Hz, 1H), 2.81-2.60 (m, 10H), 2.51-1.90 (m, 10H), 1.36-1.19 (m, 12H), 0.97 (t, *J* = 7.2 Hz, 3H).

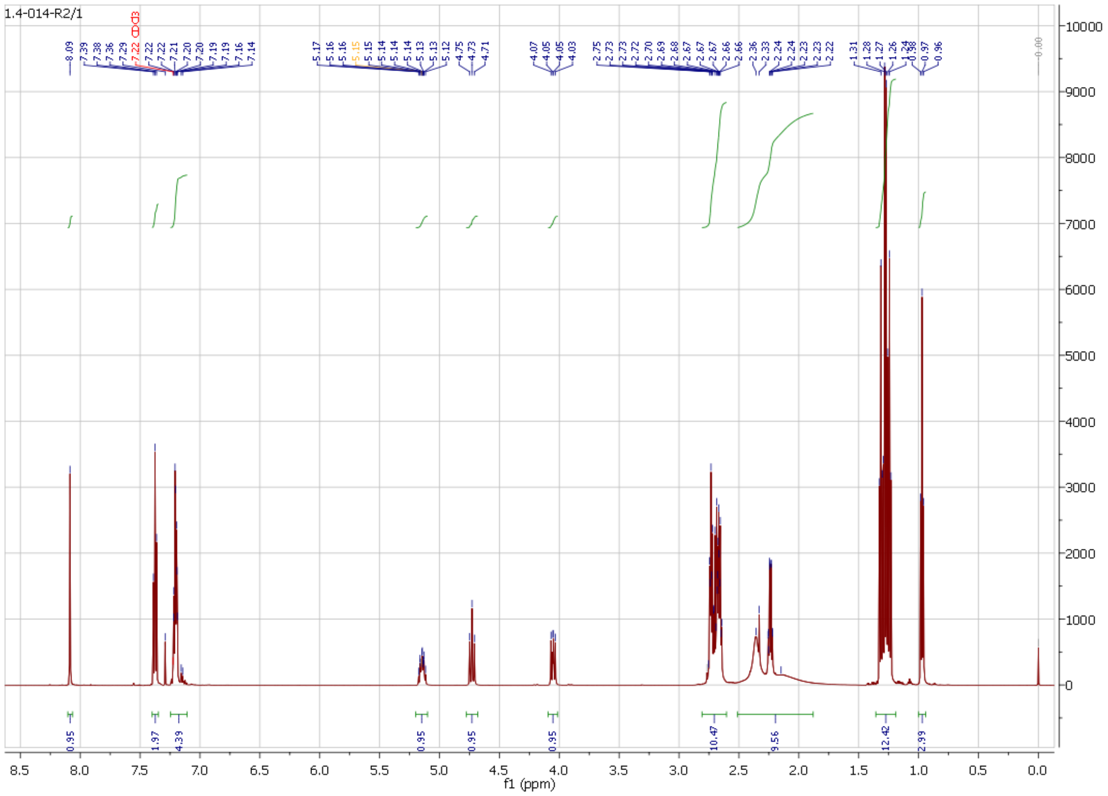

^{13}C NMR (150 MHz, CDCl$_3$) δ ppm: 158.53, 141.67, 141.27, 141.20, 140.79, 131.13, 131.08, 130.99, 130.48, 129.01, 128.20, 127.46, 127.37, 127.26, 126.72, 62.02, 59.81, 56.74, 53.00, 52.36, 52.09, 24.26, 24.22, 24.06, 23.97, 23.87, 14.92, 14.90, 14.79, 11.86.

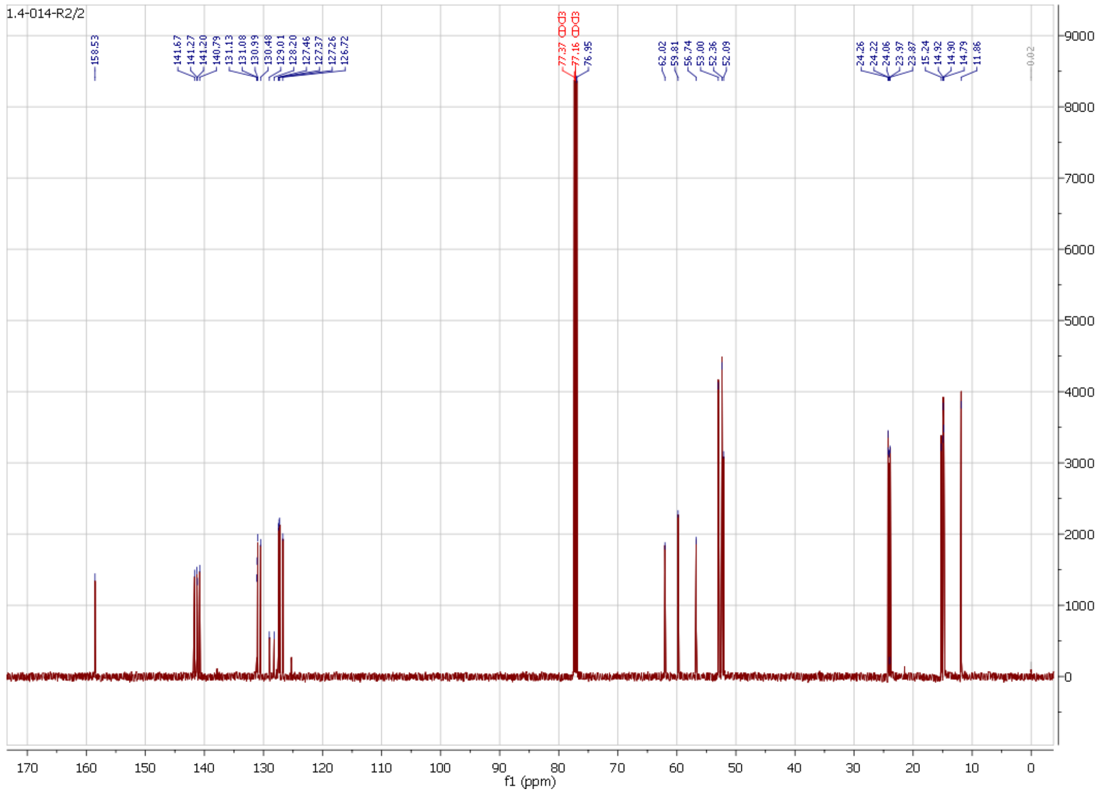

2.1.3 Dichloro(1,3-bis(2,6-diethylphenyl)-4-((4-ethylpiperazin-1-yl)methyl)imidazolidin-2-ylidene)(2-isopropoxybenzylidene)ruthenium(II) (23)

t-AmOK (1.7 M in toluene, 17.28 ml, 29.4 mmol) was added to the suspension of salt **22** in degassed toluene (210 ml). The reaction mixture was stirred for 10 min at room temperature. Then, **Ind-I** was added (24.82 g, 28.0 mmol) and the mixture was stirred for 15 min at 80 °C. Next, 1-isopropoxy-2-(prop-1-en-1-yl)benzene was added (4.93 g, 28.0 mmol), followed by copper chloride (6.10 g, 61.6 mmol) and the mixture stirred for additional 15 min. After cooling to room temperature the insoluble material was filtered off and washed with toluene. The crude product was purified using column chromatography (5% to 20% EtOAc in c-hex as eluent) affording product **23** as green powder (6.8 g, 31% yield).

^{1}H NMR (600 MHz, CD_2Cl_2) δ ppm: 16.36 (s, 1H), 7.72-7.65 (m, 3H), 7.64-7.48 (m, 7H), 7.45-7.30 (m, 3H), 6.98-6.88 (m, 2H), 6.86 (d, *J* = 7.8 Hz, 1H), 4.89 (septet, *J* = 6.0 Hz, 1H), 4.62-4.53 (m, 1H), 4.34 (t, *J* = 10.2 Hz, 1H), 4.10 (dd, *J* = 10.8, 8.4 Hz, 1H), 3.10-2.81 (m, 4H), 2.81-2.71 (m, 2H), 2.70-2.64 (m, 1H), 2.56-2.52 (m, 1H), 2.44-2.32 (m, 5H), 1.49-1.13 (m, 19H), 1.06 (t, *J* = 7.2 Hz, 3H).

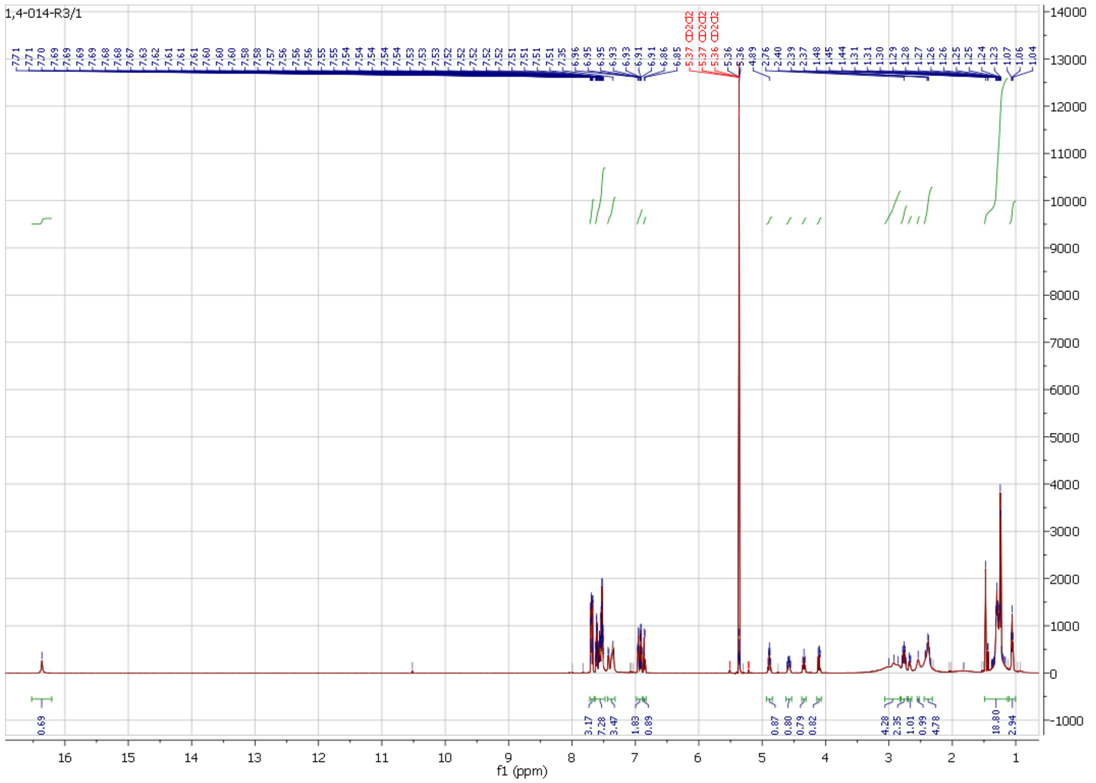

^{13}C NMR (150 MHz, CD_2Cl_2) δ ppm: 294.33 (Ru=CH), 213.37 (RuCNN), 151.99, 144.75, 144.74, 131.95, 131.88, 131.86, 128.53, 128.45, 122.23, 112.90, 75.04, 52.60, 52.11, 26.93, 21.11, 21.09, 14.38, 11.68.

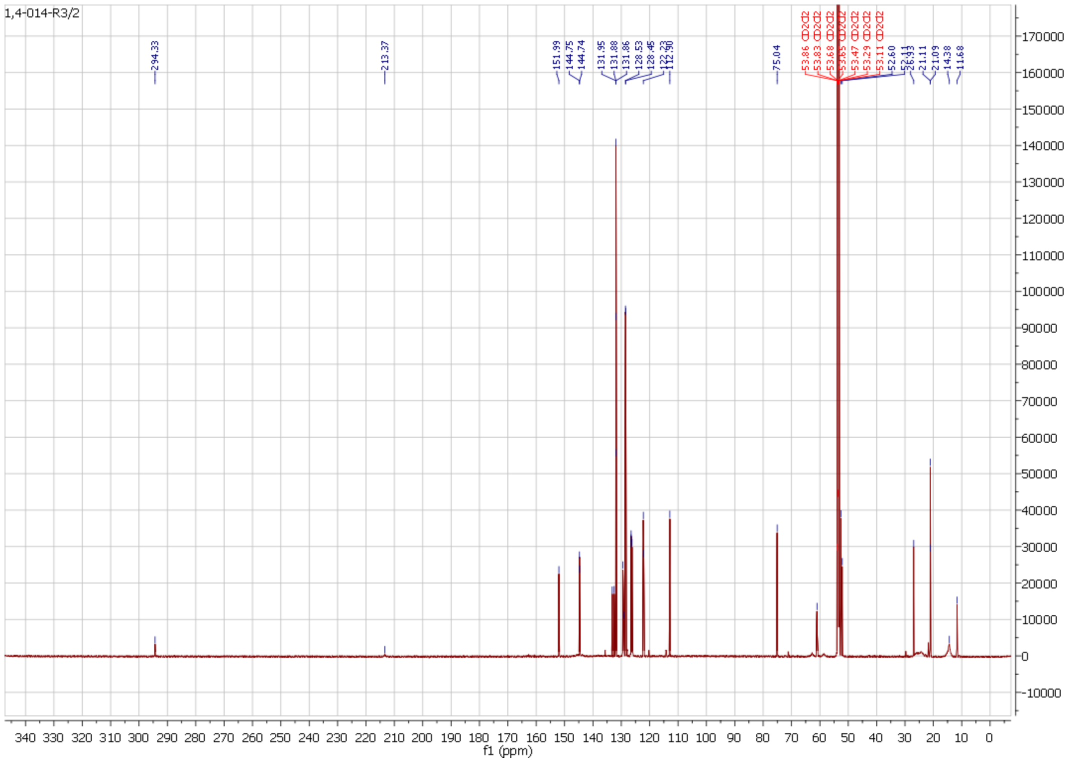

2.1.4 Dichloro(1,3-bis(2,6-diethylphenyl)-4-((4-ethyl-4-methylpiperazin-1-yl)methyl)imidazolidin-2-ylidene)(2-isopropoxybenzylidene)ruthenium(II) chloride (2a)

Complex **23** (3 g, 3.84 mmol) was placed in a pressure round-bottomed flask equipped with a magnetic stirrer and dissolved in 38.4 ml of EtOAc. Chloromethane was added as liquid (38.8 g, 768 mmol) and the solution was stirred for 20 h at 60 °C. The reaction mixture was cooled to −30 °C, the pressure flask opened and the solution slowly warmed to room temperature, allowing for evaporation of unreacted chloromethane. The, additional 30 ml of AcOEt were added, the precipitated product filtered off and washed with AcOEt. The crude product was purified using column chromatography (deactivated aluminium oxide (5% H_2O), eluent: 5% MeOH in dichloromethane) to afford product **2a** as green powder (3.24 g, 95% yield).

^{1}H NMR (600 MHz, CD_2Cl_2) δ ppm: 16.33 (s, 1H), 7.61-7.50 (m, 3H), 7.46-7.42 (m, 1H), 7.41-7.32 (m, 3H), 6.96-6.90 (m, 2H), 6.87 (d, *J* = 7.8 Hz, 1H), 4.89 (septet, *J* = 6 Hz, 1H), 4.65-4.56 (m, 1H), 4.38 (t, *J* = 10.2 Hz, 1H), 4.02 (t, *J* = 9.6 Hz, 1H), 3.72-3.66 (m, 2H), 3.59-3.55 (m, 1H), 3.36-3.30 (m, 1H), 3.23 (s, 2H), 3.10-3.01 (m, 1H), 2.94-2.67 (m, 13H), 1.41-1.10 (m, 24H).

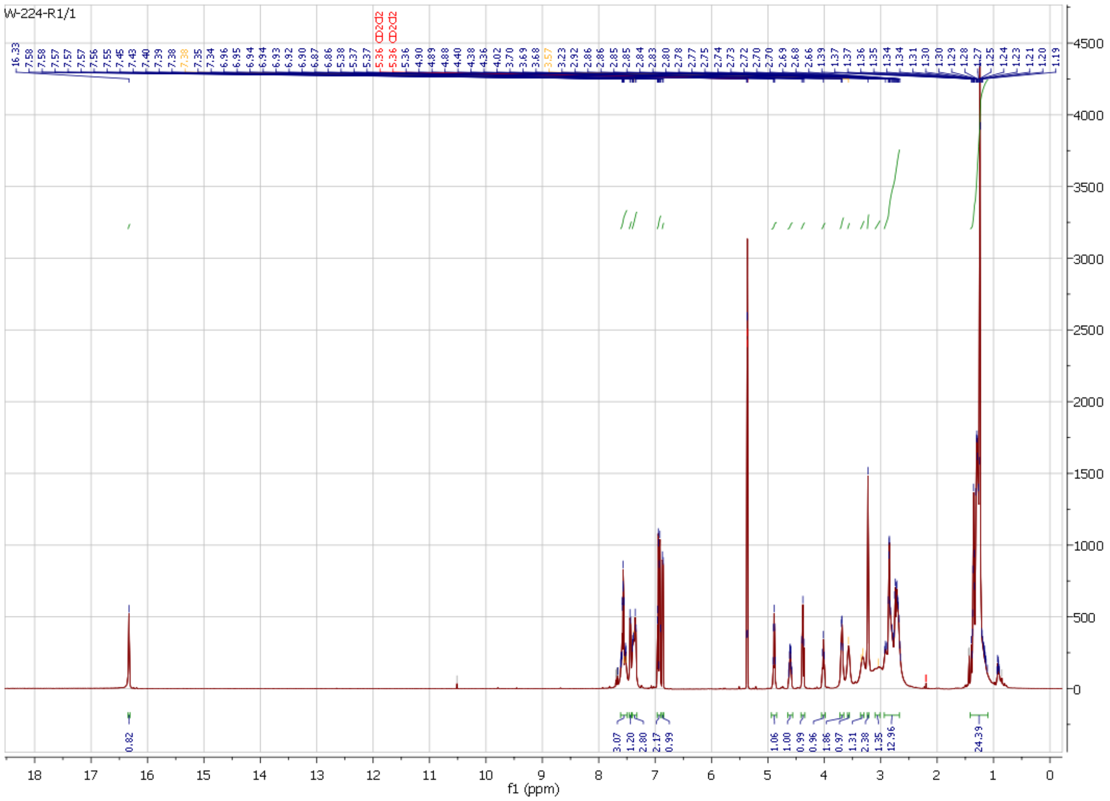

^{13}C NMR (150 MHz, CD_2Cl_2) δ ppm: 294.43 (Ru=CH), 214.42 (RuCNN), 152.07, 144.68, 144.62, 129.61, 126.54, 126.47, 126.26, 122.27, 122.13, 112.93, 75.16, 60.03, 59.67, 59.65, 53.34, 53.16, 47.11, 46.73, 21.11, 21.09, 14.21, 7.65.

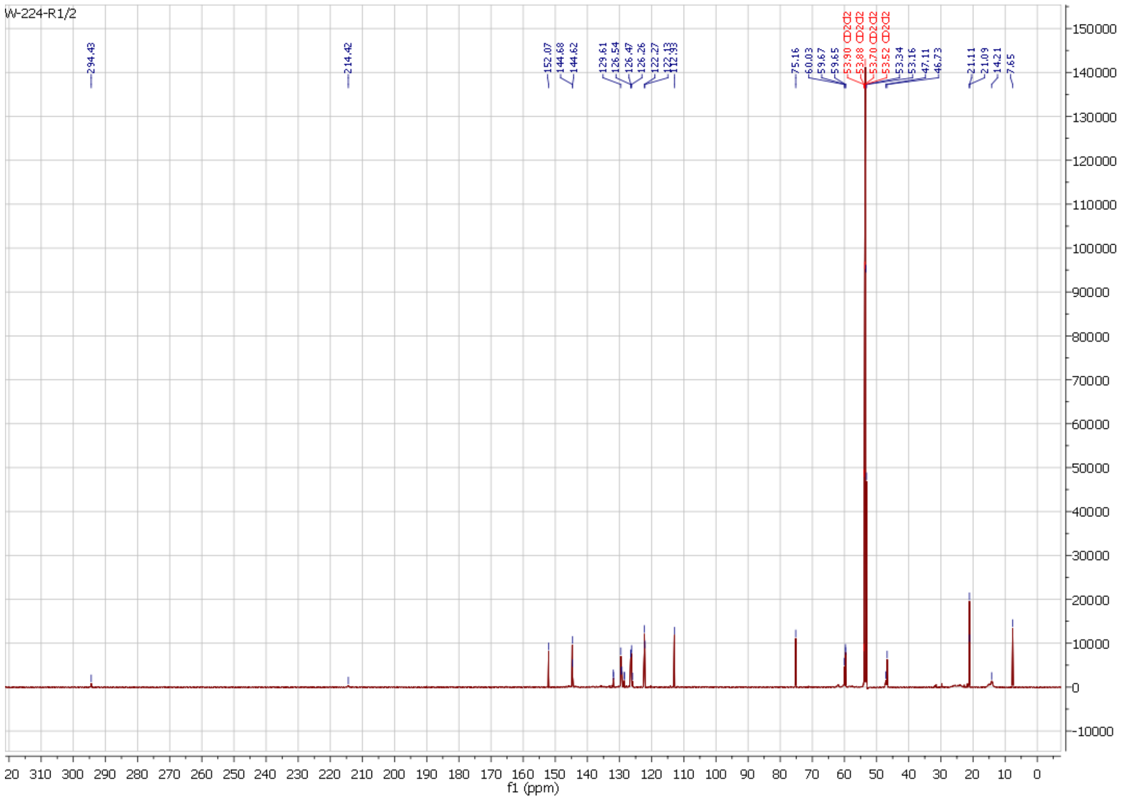

HRMS (ESI) calculated for C41H59Cl2N4ORu: 795.3109, found: 795.3120

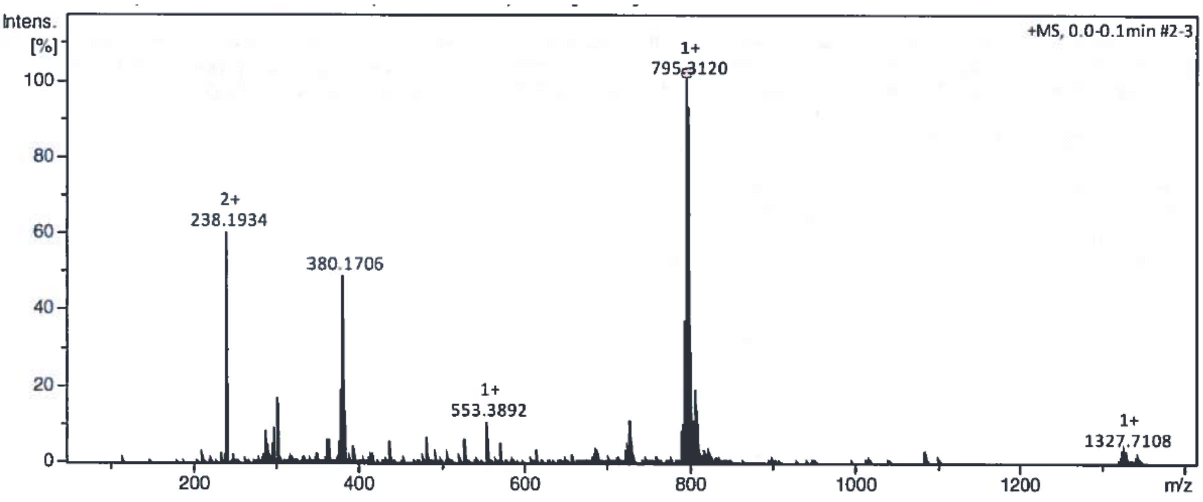

Meas. m/z	#	Ion Formula	m/z	err [ppm]	mSigma	# Sigma	Score	rdb	e— Conf	N-Rule
795.3120	1	C41H59Cl2N4ORu	795.3109	-1.4	31.4	1	100.00	14.0	odd	-

2.2 Synthesis of catalyst 2c

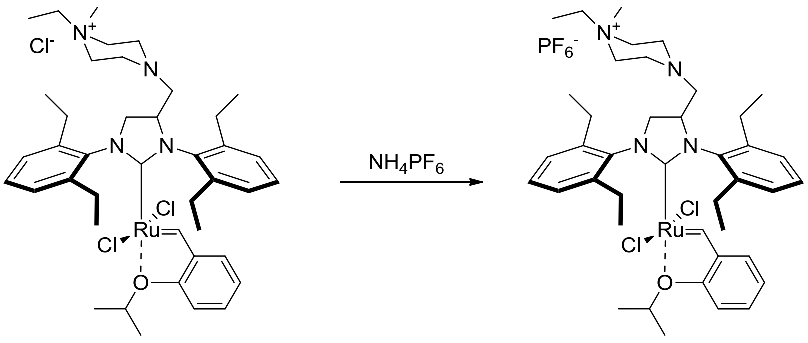

A solution of ammonium hexafluorophosphate (1.762 g, 10.81 mmol) in 15 ml of water was added under argon to the solution of catalyst **2a** (1.50 g, 1.802 mmol) in dichloromethane (25 ml). The reaction mixture was stirred at room temperature for 1 h. The aqueous phase was separated and removed. The organic layer was washed with deionized water twice and the solvents were evaporated. The catalyst was purified with column chromatography (deactivated aluminium oxide (5% H_2O), eluent: MeOH in DCM 5%), recrystallized from a DCM/pentane solvent system and dried under reduced pressure. Product **2c** was obtained as pale green powder (1.41 g, 83% yield).

^{1}H NMR (600 MHz, CD_2Cl_2) δ ppm: 16.33 (s, 1H), 7.63-7.50, (m, 3H), 7.47-7.43 (m, 1H), 7.43-7.33 (m, 3H), 6.99-6.88 (m, 2H), 6.87 (d, *J* = 8.4 Hz, 1H), 4.89 (septet, *J* = 6 Hz, 1H), 4.65-4.54 (m, 1H), 4.37 (t, *J* = 12 Hz, 1H), 4.01 (t, *J* = 9.6 Hz, 1H), 3.38-3.17 (m, 6H), 3.10-3.03 (m, 1H), 2.95 (s, 4H), 2.88-2.63 (m, 9H), 1.69 (s, 1H), 1.38-1.11 (m, 23H).

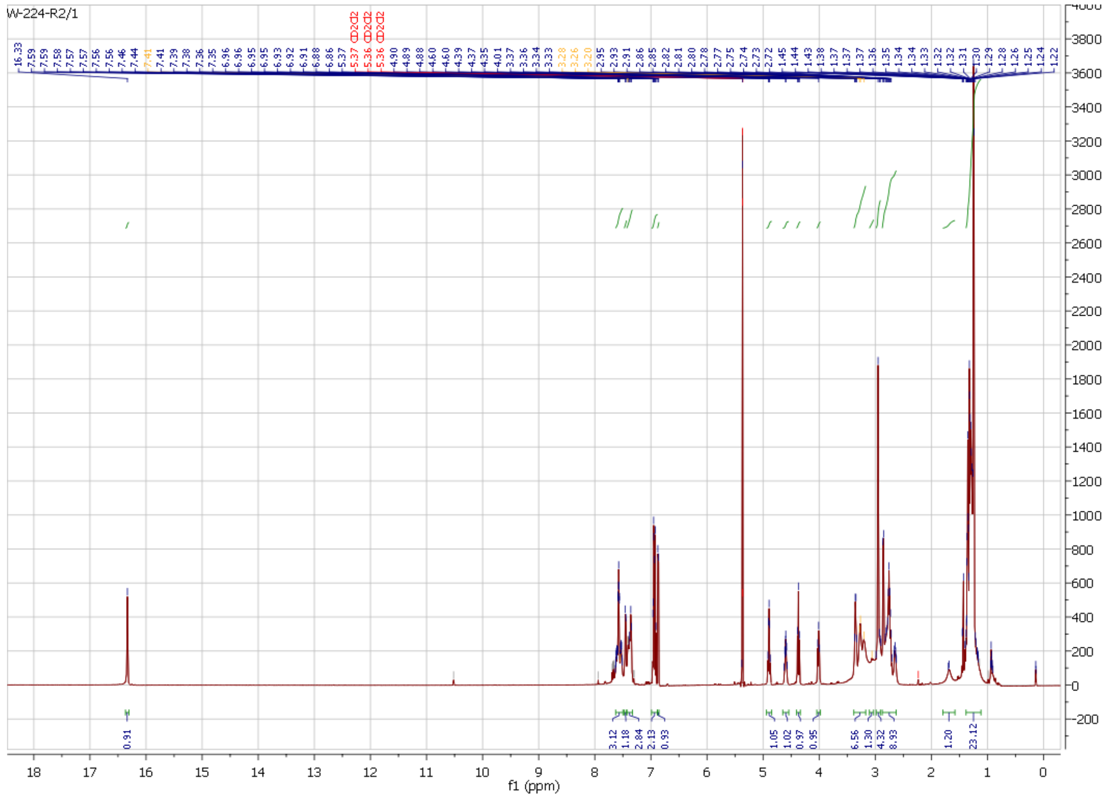

^{13}C NMR (150 MHz, CD_2Cl_2) δ ppm: 294.45 (Ru=CH), 214.48 (RuCNN), 152.08, 144.68, 144.60, 129.66, 126.58, 126.48, 126.29, 122.30, 122.15, 112.94, 75.20, 60.07, 60.04, 59.88, 53.48, 53.30, 53.12, 46.47, 29.70, 21.12, 21.10, 14.19, 7.31.

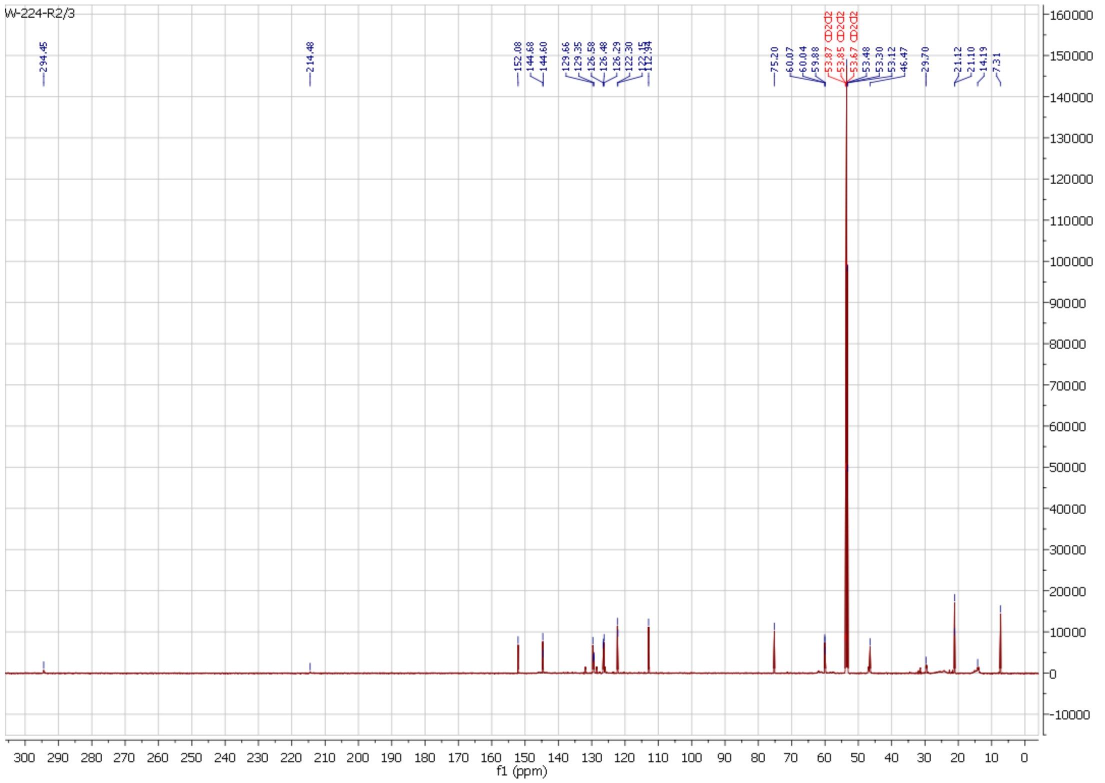

^{31}P NMR (243 MHz, CD_2Cl_2) δ ppm: 144.5 (septet, J = 711.3 Hz)

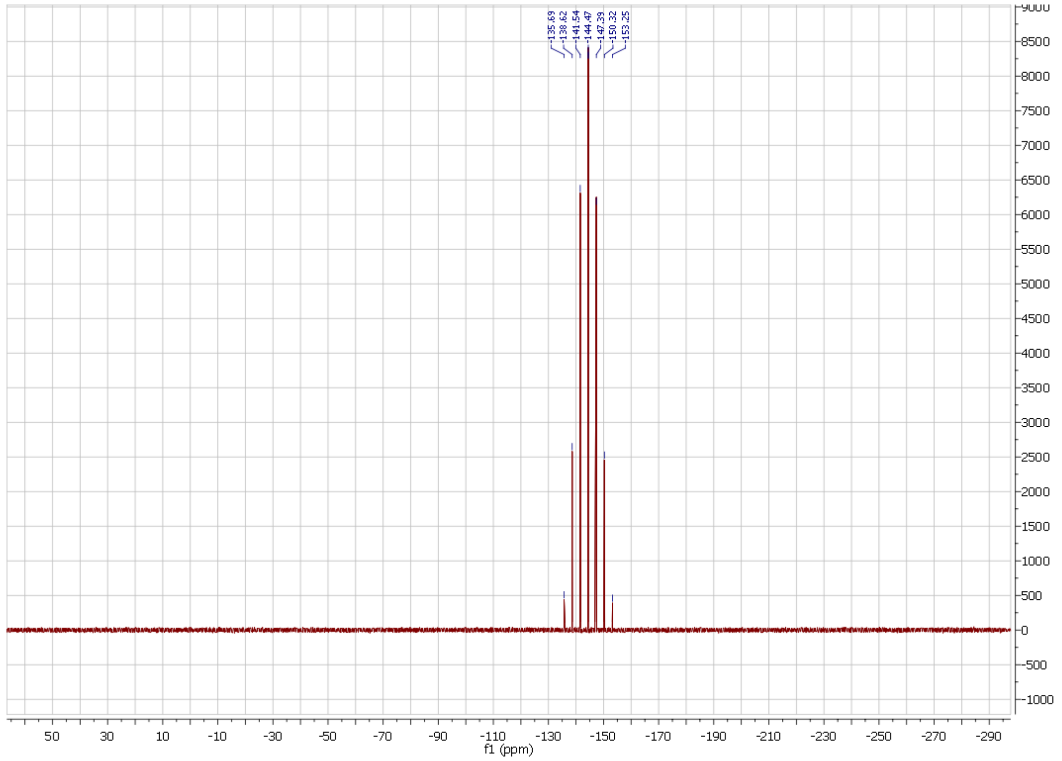

HRMS (ESI) calculated for C41H59Cl2N4ORu: 795.3109, found: 795.3116

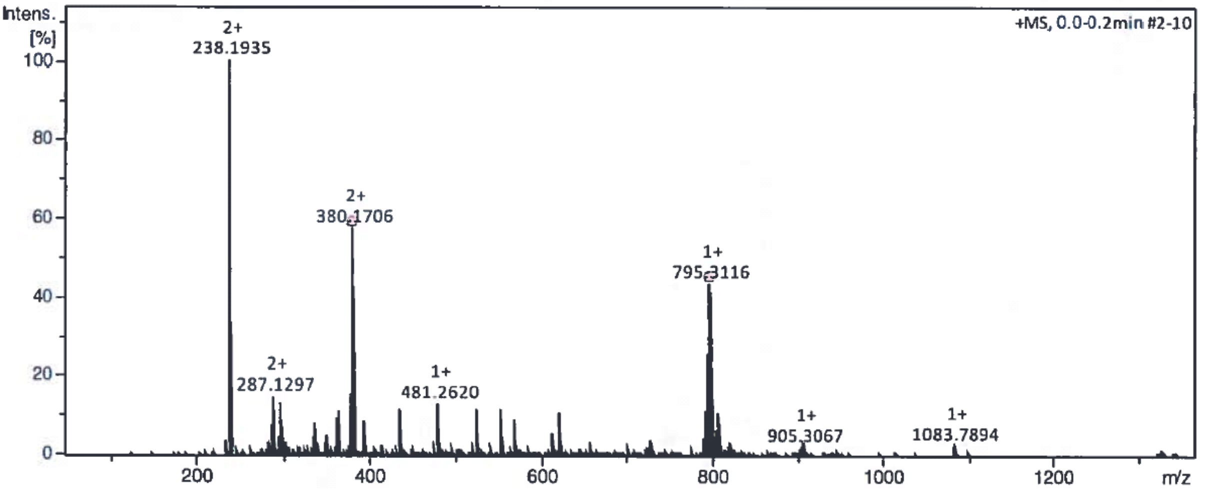

Meas. m/z	#	Ion Formula	m/z	err [ppm]	mSigma	# Sigma	Score	rdb	e— Conf	N-Rule
380.1706	1	C41H59ClN4ORu	380.1709	0.9	31.3	1	100.00	14.5	odd	-
795.3116	1	C41H59Cl2N4ORu	795.3109	0.9	27.4	1	100.00	14.0	odd	-

3. Olefin metathesis reactions

3.1 General procedure for metathesis under classical conditions

The catalyst was placed in a round-bottomed flask in a dichloromethane solution (50 µl). The solvent was evaporated under reduced pressure and the catalyst was diluted with deionized water, deuterium oxide or deuterium oxide/iPrOH mixture (*c* 0.2 M). The reaction vessel was placed in an oil bath and thermostated at 36 °C for 15 minutes. Then, the substrate (1 mmol) was added in one portion and after 2 h the reaction was quenched with ethyl vinyl ether in ethyl acetate (1 M). Products soluble in water were analysed by NMR. Lipophilic substrates and products were extracted with AcOEt or diethyl ether and analysed by GC.

3.2 General procedure for metathesis under ultrasound irradiation

The catalyst was placed in a glass tube in a dichloromethane solution (50 µl). The solvent was evaporated under reduced pressure and the catalyst was diluted with deionized water, deuterium oxide or deuterium oxide/iPrOH mixture. The reaction vessel was placed in an ultrasound water bath and thermostated at 36 °C for 15 minutes. Then, substrate (1 mmol, *c* 0.2 M) was added in one portion and sonication switched on. After 2 h the reaction was quenched with ethyl vinyl ether in ethyl acetate (1 M). Products soluble in water were analysed by NMR. Lipophilic substrates and products were extracted with AcOEt or diethyl ether and analysed by GC.

3.3 General procedure for metathesis under microwave irradiation

The catalyst was placed in a Teflon MarsXpress™ Teflon tube in a dichloromethane solution (50 µl). The solvent was evaporated under reduced pressure and the catalyst was diluted with deionized water, deuterium oxide or deuterium oxide/iPrOH mixture (*c* 0.2 M). Then, substrate (3 mmol) was added in one portion and the reaction mixture was subjected to microwave irradiation. After 2 h the reaction was quenched with ethyl vinyl ether in ethyl acetate (1 M). Products soluble in water were

analysed by NMR. Lipophilic substrates and products were extracted with AcOEt or diethyl ether and analysed by GC.

Microwave digestion system program:
Ramp: 4 min to 36 °C; hold: 120 min. Power: 30 W.

4. Synthesis and characterization of compounds 6 and 7

4.1 Synthesis of *N*,*N*-diallyl-2-(trimethylammonio)acetamide chloride (6)

2-Chloroacetyl chloride (204 mmol, 23.73 g) was placed in a round-bottomed flask and dissolved in 424 ml of dichloromethane. The solution was cooled to −15 °C and a mixture of diallylamine (204 mmol, 20 g) and trimethylamine (306 mmol, 31.2 g) was added dropwise. The solution was slowly warmed to room temperature and stirred overnight. The resulting mixture was washed with water and the organic phase was dried over anhydrous sodium sulphate and the crude product filtered through silica gel. Solvents were evaporated and the resulting product (144 mmol, 25 g) was placed in a pressure flask. Trimethylamine (158 mmol) was added as ethanol solution (4.2 M, 37.7 ml) and the reaction mixture was stirred for 20 h at 60 °C. Solvents were evaporated and the product was crystallised from diethyl ether. Solid material was filtered off, washed with diethyl ether and dried under reduced pressure. Product **6** was obtained as white powder (32 g, 68% yield).

^{1}H NMR (600 MHz, $CDCl_3$) δ ppm: 5.91-5.81 (m, 1H), 5.73-5.63 (m, 1H), 5.24-5.12 (m, 4H), 5.04 (s, 2H), 4.06 (d, *J* = 5.4 Hz, 2H), 3.92 (d, *J* = 6 Hz, 2H), 3.58 (s, 9H).

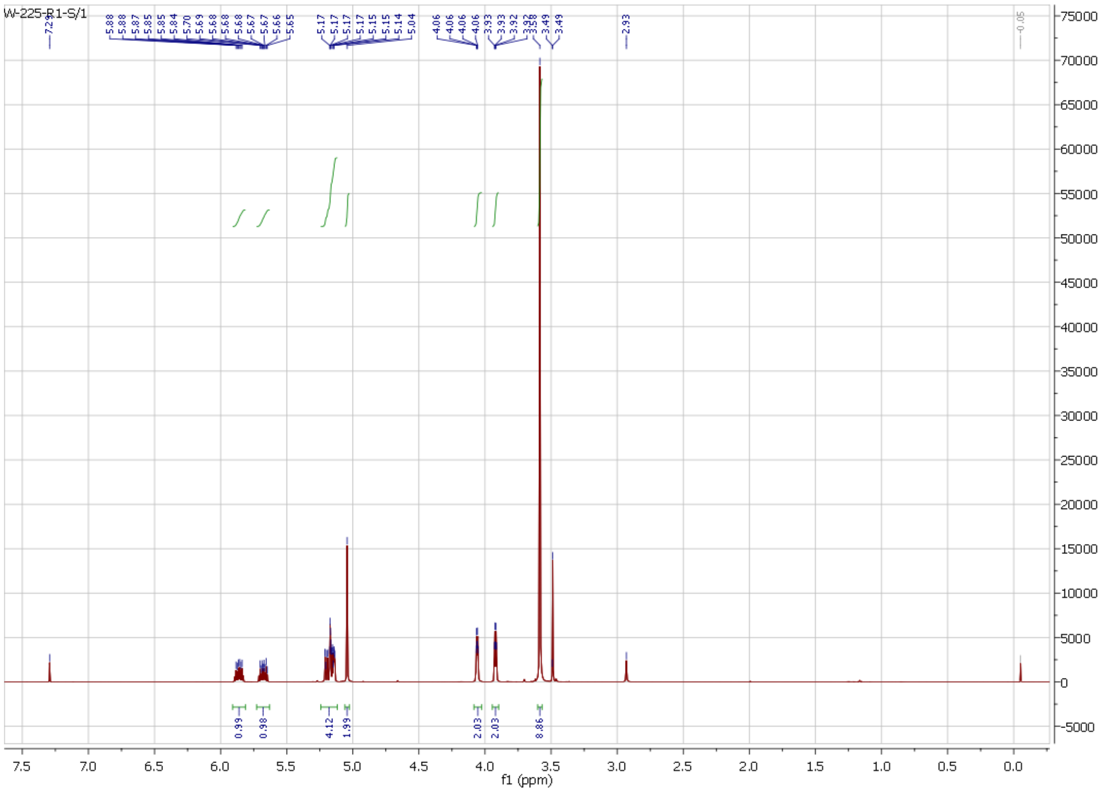

4.2 Characterisation of 2-(2,5-dihydro-1*H*-pyrrol-1-yl)-*N*,*N*,*N*-trimethyl-2-oxoethanaminium chloride (7)

^{1}H NMR (600 MHz, CDCl$_3$) δ ppm: 5.86-5.79 (m, 2H), 5.14 (s, 2H), 4.52-4.44 (m, 2H), 4.25-4.20 (m, 2H), 3.66 (s, 9H).

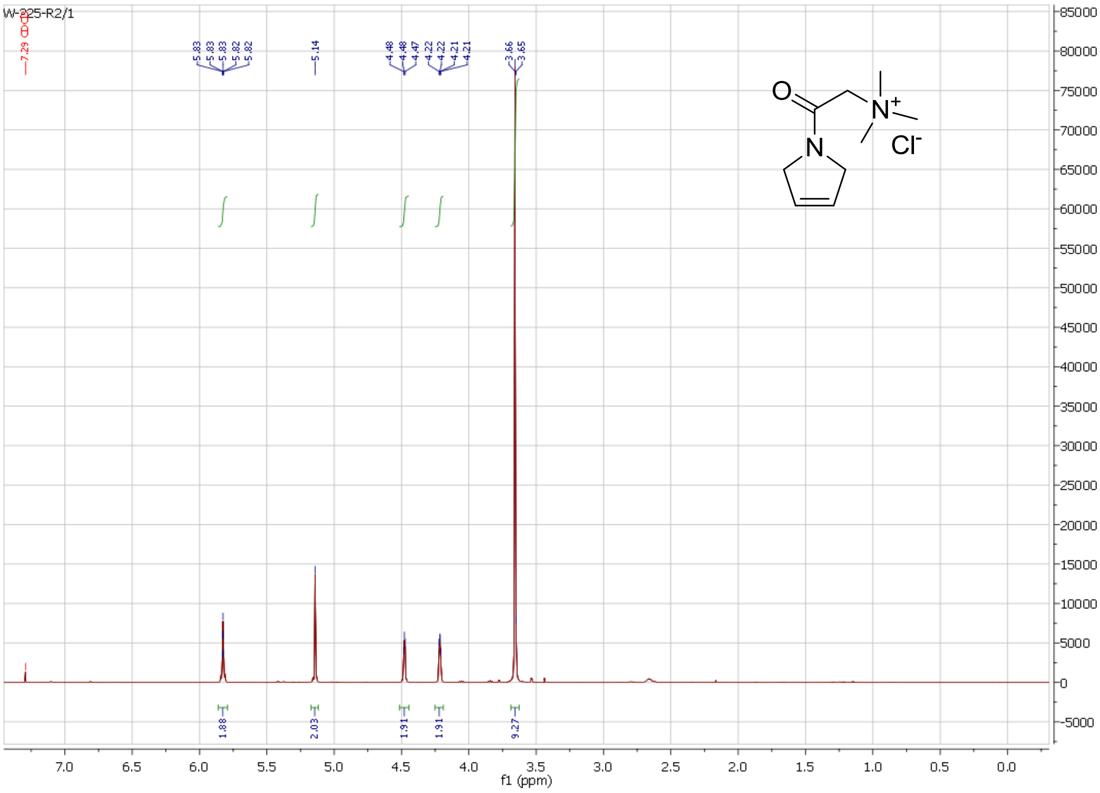

Supplement: File 1 — Experimental procedures and characterisation data for all previously unreported compounds. [file Beilstein_J_Org_Chem-15-160-s001.pdf]
